# Green Analytical Methods of Antimalarial Artemether-Lumefantrine Analysis for Falsification Detection Using a Low-Cost Handled NIR Spectrometer with DD-SIMCA and Drug Quantification by HPLC

**DOI:** 10.3390/molecules25153397

**Published:** 2020-07-27

**Authors:** Moussa Yabré, Ludivine Ferey, Abdoul Karim Sakira, Camille Bonmatin, Clotilde Fauré, Touridomon Issa Somé, Karen Gaudin

**Affiliations:** 1 ChemBioPharm Team, ARNA Laboratory, INSERM U1212, CNRS UMR 5320, 146, Rue Léo Saignat, Bordeaux University, 33076 Bordeaux, France; moussa.yabre@u-bordeaux.fr (M.Y.); ludivine.ferey@u-bordeaux.fr (L.F.); camille.bonmatin@gmail.com (C.B.); clotilde.faure@etu.u-bordeaux.fr (C.F.); 2 Laboratoire de Toxicologie, Environnement et Santé (LATES), Université Joseph Ki-Zerbo, Ouaga 03 BP 7021, Burkina Faso; karim_sakira@yahoo.fr (A.K.S.); tsome@ulb.ac.be (T.I.S.)

**Keywords:** antimalarial artemether-lumefantrine, green chromatography, accuracy profile, handheld NIR spectrometer, non-destructive analysis, drug falsification detection, PCA and DD-SIMCA, quality control

## Abstract

Two green analytical approaches have been developed for the analysis of antimalarial fixed dose tablets of artemether and lumefantrine for quality control. The first approach consisted of investigating the qualitative performance of a low-cost handheld near-infrared spectrometer in combination with the principal component analysis as an exploratory tool to identify trends, similarities, and differences between pharmaceutical samples, before applying the data driven soft independent modeling of class analogy (DD-SIMCA) as a one-class classifier for proper drug falsification detection with 100% of both sensitivity and specificity in the studied cases. Despite its limited spectral range and low resolution, the handheld device allowed detecting falsified drugs with no active pharmaceutical ingredient and identifying specifically a pharmaceutical tablet brand name. The second approach was the quantitative analysis based on the green and fast RP-HPLC technique using ethanol as a green organic solvent and acetic acid as a green pH modifier. The optimal separation was achieved in 7 min using a mobile phase composed of ethanol 96% and 10 mM of acetic acid pH 3.35 (63:37, *v*/*v*). The developed method was validated according to the total error approach based on an accuracy profile, was applied to the analysis of tablets, and allowed confirming falsified drugs detected by spectroscopy.

## 1. Introduction

The quality control (QC) of pharmaceutical products is a key issue in the medicine supply chain, as it guarantees drug reliability before consumption. It allows fighting against substandard and falsified drugs which present a serious threat of public health worldwide, but particularly in developing countries where monitoring systems are less effective. According to the World Health Organization (WHO), falsified medicines are defined as products that deliberately or fraudulently misrepresent their identity, composition or source and are not to be confused with counterfeit medicines, the latter term being associated with the protection of intellectual property rights [1].

QC is conventionally performed according to pharmacopeias in which methods are most often long and use harmful reagents for the technical staff, health, and environment. Greening analytical methods have received increasing attention and acceptance among researchers with the aim of minimizing the environmental impacts and improving analysts’ health safety [2,3,4]. The green analytical chemistry (GAC) involves efforts to avoid the use of toxic or hazardous chemicals and their replacement by more ecofriendly ones, together with an important reduction in the consumption of energy, reagents, and solvents, proper management of analytical waste, and miniaturization of analytical devices [5]. From a green point of view, analytical techniques can be divided into two groups. The first group includes direct analytical techniques which are inherently green [6]. This is the case of vibrational spectroscopic techniques such as near-infrared (NIR) spectroscopy which are non-destructive and require neither reagents nor a sample preparation step. This technique associated to chemometric tools are nowadays more and more employed for both qualitative (product identification and particularly detection of falsified drugs) and quantitative (determination of drug content) analysis in a pharmaceutical quality control [7,8,9]. However, the high cost of instruments classically commercialized limits their use, particularly in resource limited laboratories. Fortunately, some innovative handheld and low-cost NIR spectrophotometers (less than 1000 EUR) have recently appeared on the market. In addition to their low cost, these portable devices can offer a promising performance comparable to bench-top instruments [10,11]. The second group of analytical techniques concerns methods that require reagents and sample preparation, and for which different modifications must be applied to be conformed as much as possible to GAC concepts [12]. One example is HPLC which is the most used analytical tool in a pharmaceutical analysis in QC and commonly use high amounts of hazardous organic solvents such as acetonitrile [13,14]. Greening HPLC methods can be solved by substituting the commonly used organic solvents by greener ones [15,16,17]. Ethanol is one of the best green alternative solvents because of its low toxicity, biosourcing, biodegradability, and safety of handling by operators [18].

The artemether and lumefantrine (AL) association is one of the WHO-recommended artemisinin-based combination therapies to treat uncomplicated *Plasmodium falciparum* malaria. AL formulations are part of the WHO Model lists of Essential Medicine [19]. However, it is also one of the most falsified class of antimalarial drugs in developing countries [20]. In the International Pharmacopeia [21], AL quantitative determination is carried out by RP-HPLC. However, this analytical technique has green issues since it is long (55 min) and employs acetonitrile as an organic solvent and an ion pairing agent (hexanesulfonate) to improve the lumefantrine peak shape. Moreover, ion-pair reagents most often drastically increase the equilibration time of the chromatographic column, leading to a high consumption of mobile phases and long analysis times. In the literature, there are some publications about the simultaneous HPLC analysis of AL in formulations [22,23,24,25,26,27]. However, they have not been developed according to the green analytical chemistry principles. Since AL formulations represent one of the most falsified antimalarial drugs, it is also of interest to have a fast-qualitative screening tool based on NIRS associated with the principal component analysis (PCA) and data driven soft independent modeling of class analogy (DD-SIMCA) allowing to check the presence of the active pharmaceutical ingredient (API) and to authenticate a brand name, before performing further quantitative analysis.

In this report, for the first time two green analytical approaches have been combined: (a) For proper falsification detection in solid dosage forms of antimalarial artemether-lumefantrine in a fixed-dose combination. This is achieved by using a non-destructive analysis based on a low-cost handheld NIR spectrometer in association with PCA and DD-SIMCA as a screening tool to identify specifically a medicinal brand name and to detect quickly suspicious falsified medicines; (b) for quantitative analysis based on a green and fast RP-HPLC technique using ethanol as a green organic solvent and acetic acid as a green pH modifier. 

## 2. Results and Discussion

### 2.1. Near-Infrared Analysis

The aim was to evaluate the potential of a low-cost (less than 1000 EUR) handheld NIR spectrophotometer (NIR-S-G1, from Innospectra) as a screening tool for falsified drug identification. For this task, DD-SIMCA was used as a classification model which performed a PCA. This one-class classifier is a useful chemometric tool for the verification of product identity and for the detection of falsified drugs [28,29].

#### 2.1.1. Data Acquisition and Preprocessing

**Figure 1:**
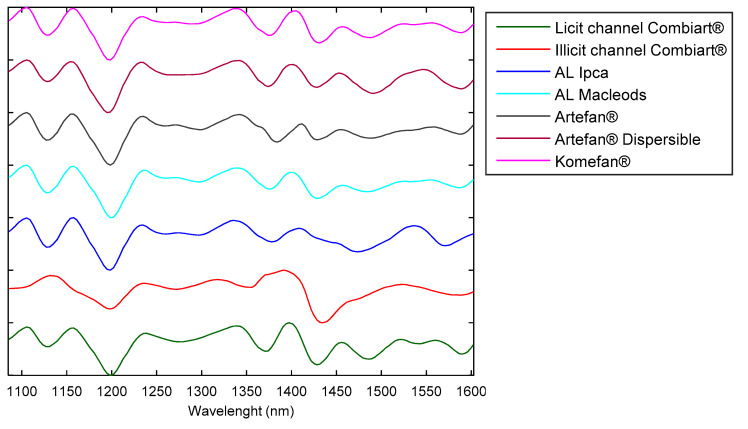
Artemether-lumefantrine (AL) preprocessed spectra in the 1085–1601 nm range.

The analysis was performed on artemether-lumefantrine tablets from different brands collected in local pharmacies (Table 1). Moreover, some suspicious designated Combiart samples from the illicit sales channel has been analyzed. The spectrophotometer allows acquiring spectra in the 900–1700 nm region. Tablet samples were directly scanned through their blister and spectra of ten tablets per batch have been recorded for each formulation. Therefore, a total of 270 spectra was acquired. Spectral data have been preprocessed to improve the signal-to-noise ratio. First, a Savitzky-Golay (SG) smoothing and differentiation filter (second-degree polynomial and second derivative) was applied to remove noise and baseline signals. Second, a multiplicative scatter correction (MSC) was applied to the smoothed and differentiated signals. The chemometric analysis was carried out on the spectral range between 1085 and 1601 nm because under 1085 nm the area was found non-informative while upper 1601 it was found less reproducible and noisier. Preprocessed mean spectra are presented in Figure 1.

#### 2.1.2. PCA

**Figure 2:**
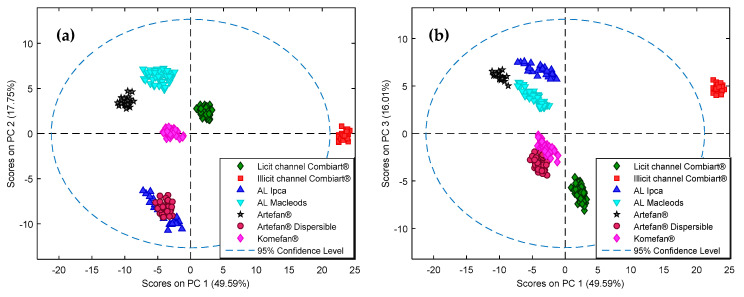
Results of the principal component analysis (PCA) applied on the preprocessed data. (**a**) PC1-PC2 score plot. (**b**) PC1-PC3 score plot.

A PCA was performed on the acquired and preprocessed spectra to have a visualization of the repartition of the dataset. In fact, PCA is a useful unsupervised model to enhance differences and similarities between the spectra, allowing the detection of underlying clusters [8,9]. The PC1-PC2 and PC1-PC3 score plots are presented in Figure 2. The three first PCs described more than 80% of variability. Along the PC1 score, samples of the suspicious designated Combiart^®^, from the illicit sales channel, were far from samples of the licit channel Combiart^®^ and other products, being outside the 95% confidence level. These samples of the illicit channel seemed to be falsified. The PC1-PC2 score plot allowed the distinction of four AL products (Combiart^®^, Komefan^®^, Artefan^®^, and AL Macleods) between each other and from the two products Artefan^®^ dispersible and AL Ipca which were not separated from each other. However, on the PC1-PC3 score plot these two products could be distinguished. Even if formulations from pharmacies can contain the correct active pharmaceutical ingredients, they could be differentiated by PCA. This can be explained by the fact that the analyzed medicines may not have the same nature and composition of excipients and that NIR spectra are sensitive not only to chemical properties but also to physical properties.

#### 2.1.3. DD-SIMCA Analysis

Based on the results obtained with the PCA, a class modeling tool such as SIMCA in its data driven (DD) version was used to confirm the ability of the handheld device to identify specifically a brand name. The DD-SIMCA model is described in Section 3.2.3. Models were built for the formulations for which at least five batches have been collected (licit Combiart^®^, Komefan^®^, and AL Macleods). Therefore, three target classes were modelized. 

**Figure 3:**
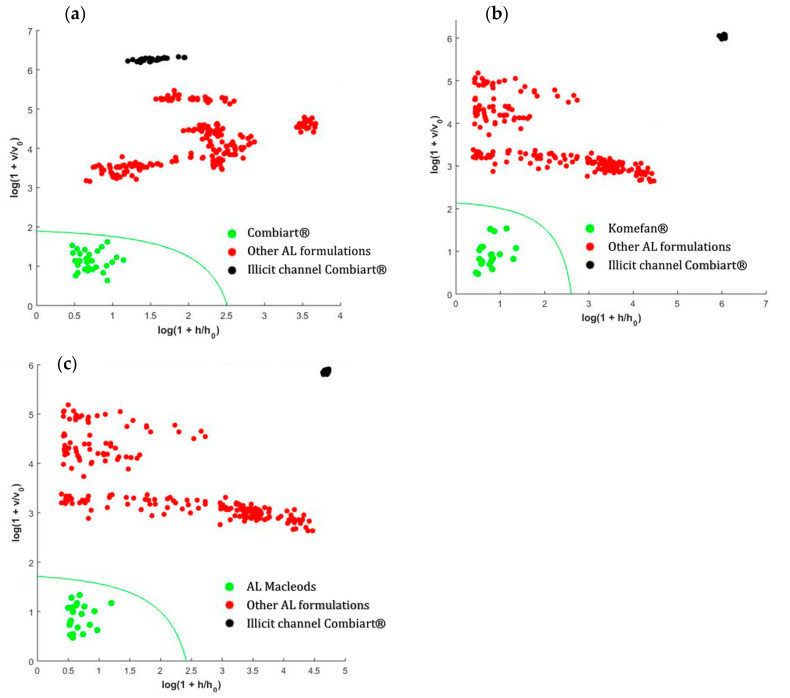
Data driven soft independent modeling of class analogy (DD-SIMCA) plots. (**a**) Combiart (two PCs, α = 10^−6^). (**b**) Komefan (two PCs, α = 10^−5^). (**c**) AL Macleods (two PCs, α = 10^−4^).

For each class, the spectra of three batches were used as a training set to build the SIMCA models and the remaining batches were used as a test set to evaluate the model sensitivity. The other AL formulations were employed to mimic high quality fake drugs and test model specificity. Falsified AL Combiart^®^ bought in the illicit sale channel was also used to prove model specificity. For each class, model parameters (number of PCs and significance level α) were optimized in a sequential way. Results of DD-SIMCA models built for the three brand products are shown in Figure 3. The DD-SIMCA models allowed an identification specific brand identification with 100% of both sensitivity and specificity in the studied cases. These results showed that despite the limited spectral range and low resolution (10 nm) of this low-cost spectrophotometer, it offers a promising qualitative performance for detection of falsified drugs.

For a better interpretation of NIR results, a green and fast HPLC technique has been developed and used to analyze AL tablets.

### 2.2. HPLC Analysis

#### 2.2.1. Method Development

**Figure 4:**
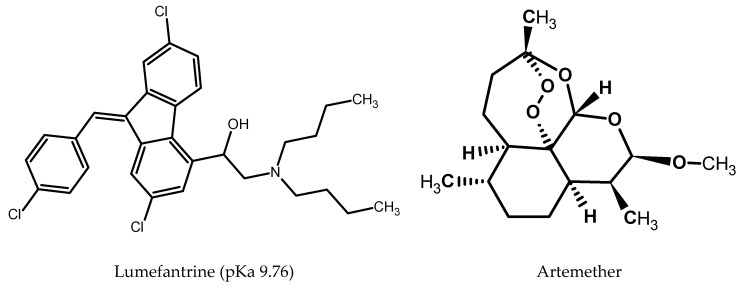
Chemical structures of lumefantrine and artemether.

With the objective to develop a green HPLC technique, ethanol, which is one of the best green solvents, was selected as an organic solvent. Considering the high viscosity of ethanol mobile phases, the flow rate was set at 0.5 mL/min to avoid a high column backpressure. Artemether is a neutral compound with weak chromophores while lumefantrine has basic properties and greatly absorbs in UV (Figure 4). Due to the poor absorption of artemether, detection was done at 210 nm. A pH selection of the mobile phase is required only for lumefantrine since artemether is neutral. To limit the lumefantrine interaction with residual silanols a pH value higher than its pKa would be necessary. However, this will limit the stable stationary phases at pH 11. Therefore, the pH of the aqueous part of the mobile phase was fixed using 10 mM of acetic acid (pH 3.35) which is a benign and low toxic pH modifier to respect the green analytical principles. At this pH, lumefantrine is in its ionic form with a positive charge, which could interact with the free silanols of the stationary phase, leading to peak tailing. Therefore, column testing was carried out regarding selectivity and peak shape of the positively charged basic compound. An initial mobile phase containing EtOH 96% and 10 mM of acetic acid aqueous solution, pH 3.35 (60:40, *v*/*v*) was used. Considering that the amount of lumefantrine was six times higher than the artemether amount in tablets, a mixture of both molecules at 0.1 mg/mL for artemether and 0.6 mg/mL for lumefantrine were initially used. However, it was found that the lumefantrine peak was asymmetrical at this concentration (As > 2.5), whatever the column. The acceptable peak shape for lumefantrine was reached at a concentration under 0.020 mg/mL corresponding to a concentration of 0.0033 mg/mL of artemether in respect to the formulation ratio. Unfortunately, artemether could not be detected at such low concentration due to its lack of chromophore even at 210 nm. Consequently, two solutions were therefore prepared for the method optimization. The first one was a mixture of artemether and lumefantrine at 0.1 and 0.6 mg/mL, respectively, to be able to detect artemether with a signal-to-noise ratio above 70 and to be able to assess the retention and separation quality between these two compounds. The second one was composed of lumefantrine at 0.012 mg/mL to be able to observe a satisfactory peak shape for its quantification.

**Figure 5:**
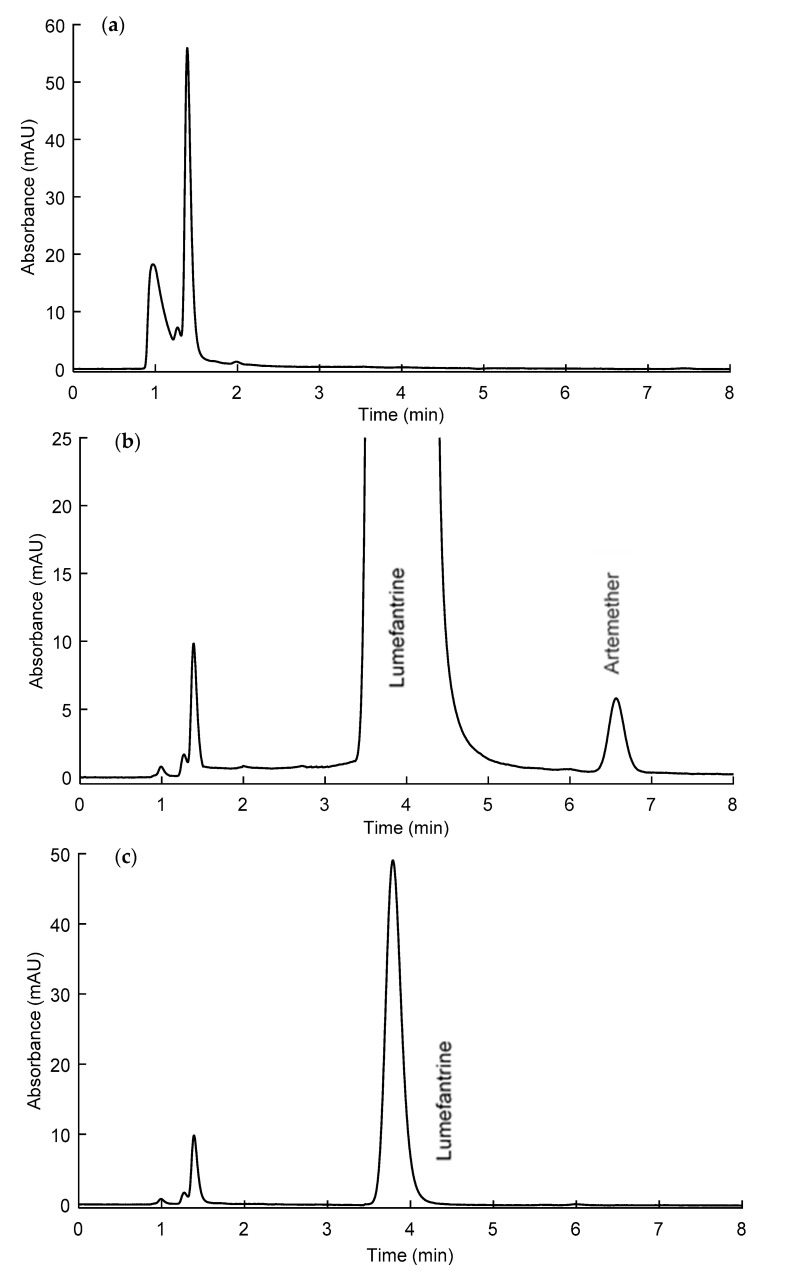
Chromatograms. (**a**) Excipient mixture, (**b**) artemether and lumefantrine at 0.1 and 0.6 mg/mL, respectively. (**c**) Lumefantrine at 0.012 mg/mL. Column: Symmetry C18, mobile phase: Ethanol 96%, and 10 mM of acetic acid pH 3.35 (63:37, *v*/*v*), 0.5 mL/min, 210 nm.

The chromatographic parameters mainly retention factors and symmetry factors were evaluated on the three columns using a mobile phase composed of ethanol 96% and 10 mM of acetic acid (60:40, *v*/*v*). The results are presented in Table 2. In these conditions, artemether and lumefantrine were satisfactorily retained on the three columns with retention factors higher than 2, as required for drug quality control. The J’sphere ODS-H80 column was the most retentive, while the XTerra one presented a poor symmetry factor for lumefantrine. The Symmetry C18 column appeared the best choice in terms of retention and peak shape compared to the two other columns. It was therefore selected for method development. Using this column, different proportions of mobile phase solvents were evaluated to reduce the analysis time while maintaining a sufficient retention and resolution between artemether and lumefantrine peaks. A mobile phase composed of ethanol 96% and 10 mM of acetic acid pH 3.35 (63:37, *v*/*v*) allowed a minimal retention factor of two, a resolution of six, and a run time under 7 min (Figure 5). These conditions were therefore adopted as the final mobile phase composition.

**Figure 6:**
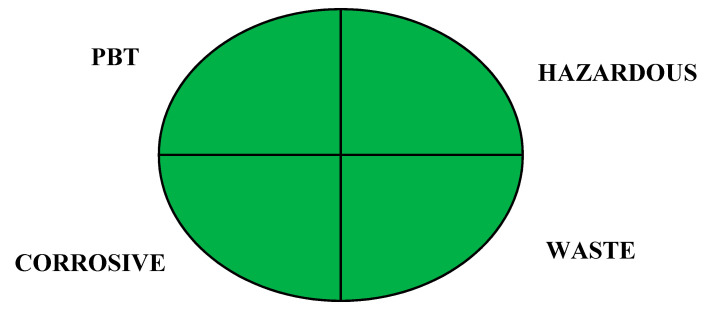
National Environmental Methods Index (NEMI) greenness profile of the developed method. Water and ethanol used in the method are neither defined as persistent, bio accumulative, and toxic (PBT) nor hazardous by the Environmental Protection Agency’s (EPA’s) Toxic Release Inventory. The pH of the mobile phase is 3.35, i.e., not corrosive, and the waste generation is <50 g/sample.

The greenness of the developed method was assessed using two different tools: The National Environmental Methods Index (NEMI) [6] and the Analytical Eco-Scale [30]. NEMI labeling results in an easy-to-read pictogram including four terms: PBT (persistent, bio accumulative, and toxic), Hazardous, Corrosive, and Waste. Each term is colored green or blank depending on whether this particular criterion is fitting or not. The analytical Eco-scale is a more quantitative approach, based on subtracting penalty points from a total of 100, considering the amount and hazard of reagents, energy consumption, occupational hazards, and amount of waste generated. A NEMI pictogram with the four terms colored in green (Figure 6) and an Eco-scale score of 94 (Table 3), allowed confirming the green character of the developed method. 

#### 2.2.2. Method Validation

The developed analytical method based on the RP-HPLC technique with a classical univariate calibration was validated to assess its ability to achieve the quantitative determination of artemether and lumefantrine in tablets according to ICH Q2(R1) [31]. Specificity, accuracy, repeatability, intermediate precision, and linearity must be established in the case of validation of the quantitative determination method. Method specificity was assessed through the injection of commonly used tablet excipients. Validation of the developed method was carried out during three days with three replicates for each concentration level. The accuracy, repeatability, intermediate precision, and linearity were assessed by the accuracy profile approach using the concept of total error (bias + standard deviation) based on the β-expectation tolerance intervals. [32,33]. The β-expectation tolerance interval defines an interval in which it is expected that each future result will fall with a defined probability β [34]. By joining the upper tolerance limits on the one hand and the lower tolerance limits on the other hand, the method defines an accuracy profile. If this profile stays within the acceptance limits set according to the needs of the final user or to regulatory expectations, the method can be considered as valid. The accuracy profile methodology is fully compliant to the ICH Q2 requirements [31].

The individual injection of all excipients and in the mixture showed no interference with the peaks of artemether and lumefantrine (Figure 5a). Therefore, the method specificity was demonstrated. 

**Figure 7:**
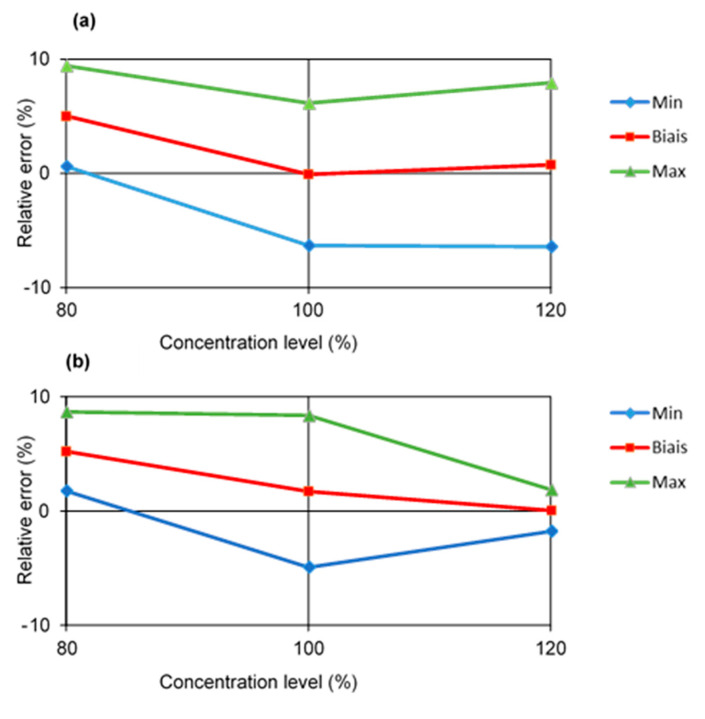
Accuracy profile. (**a**) Artemether. (**b**) Lumefantrine.

The regression model between the chromatographic pic area and the concentration of artemether or lumefantrine was studied using the 100% level of the calibration set. This allowed to back calculate concentrations of the validation set (80–120%) composed of reconstituted form samples and assess the linearity, the relative bias, the repeatability, the intermediate precision, and the β-expectation tolerance intervals at a 90% probability level. Acceptance limits were fixed at ±10% according to the International Pharmacopeia. The validation results are shown in Table 4. The linearity criterion was assessed by fitting linear regression models between the back calculated concentrations and the introduced concentrations over the dosing range. Slopes close to 1 of the regression models and R^2^ values higher than 0.99 demonstrated the good linearity of the developed method. The relative standard deviation (RSD%) values for repeatability and intermediate precision were below 4%, indicating an acceptable precision of the method. The trueness of the method was also found satisfactory since the relative biases were below 6% at each concentration level, in compliance with the acceptance limits of ±10%. The β-expectation tolerance intervals at a 90% probability level were within the acceptance limits (±10%) for each concentration level, indicating that the method was able to provide accurate results over the concentration range for each compound. The accuracy profiles are shown in Figure 7. The lower and upper limits of quantification (LLOQ and ULOQ) correspond to the dosing range for each compound: 0.08 and 0.12 mg/mL for artemether, 0.0096 and 0.0144 mg/mL for lumefantrine. 

#### 2.2.3. Tablets Analysis

The developed and validated HPLC method was used to analyze the 27 AL samples. All samples bought in the licit channel contained both artemether and lumefantrine in the adequate amount between 94% and 106% (Table 5). As expected from the NIR analysis, no active pharmaceutical ingredient was found in samples of Combiart^®^ from the illicit sales channel, confirming that they were falsified. 

**Figure 8:**
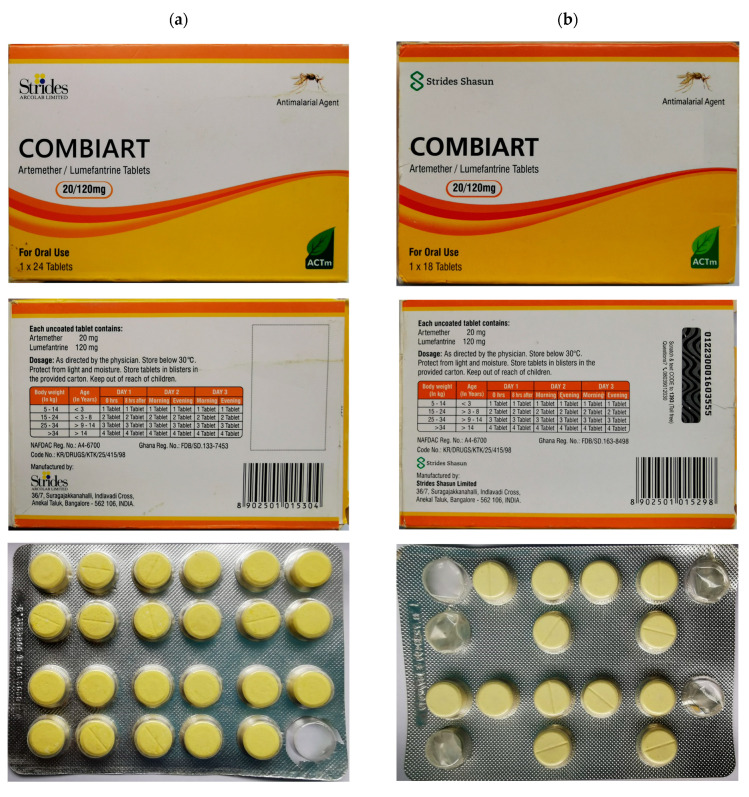
Representative photos of falsified (**a**) versus genuine (**b**) Combiart^®^ samples.

Regarding the secondary packaging, some differences can be noted for the brand logo and the batch identification (Figure 8). In the primary packaging, the number and arrangement of the tablets are also different. However, for a patient, these differences are not obvious and difficult to identify if they do not know the genuine product. In addition, the color of Combiart^®^ tablets is due to the presence of lumefantrine which is a yellow powder. No excipient in this formulation is yellow. Therefore, the falsified product found was really intended to be fake.

## 3. Materials and Methods

### 3.1. Chemicals and Reagents

The artemether and lumefantrine United States Pharmacopeia (USP) reference standards (>99%) were supplied from Sigma-Aldrich (St Louis, MO, USA). Ethanol (EtOH) 96% HPLC grade and acetic acid HPLC grade (≥99.9%) were purchased from VWR chemicals (VWR International, Fontenay-sous-Bois, France). The HPLC grade water (minimum resistivity of 18.2 MΩ) was produced in house by the ELGA Millipore system (Veolia, Saucats, France). Tablet excipients (microcrystalline cellulose, lactose, maize starch, hypromellose, croscarmellose sodium, crospovidone, povidone, talc, magnesium stearate, colloidal silica, polysorbate 80, sodium lauryl sulfate) were kindly provided by LTPIB (Laboratoire de Technologie Pharmaceutique Industrielle de Bordeaux, France). Artemether-lumefantrine (AL) 20–120 mg tablets were collected in local pharmacies in Burkina Faso and Togo. Moreover, some suspected designated Combiart samples were bought from illicit street vendors (Table 1).

### 3.2. Near-Infrared Analysis

#### 3.2.1. Instrumentation

NIR-S-G1 is a low-cost handheld dispersive spectrophotometer (less than 1000 EUR) from Innospectra (Herrsching, Germany). It is a very compact instrument (136 g of weight, 82 × 63 × 40 mm of dimensions) which can operate autonomously using batteries. It is provided with wired USB and Bluetooth wireless connections to be driven by computers, tablets, or cell phones. NIR-S-G1 allows monitoring the 900–1700 nm near-infrared spectral region with a nominal resolution of 10 nm. With two integrated tungsten halogen lamps, it analyzes the diffuse reflection of a sample surface through a scratch-resistant sapphire window. Light in the 900–1700 nm range is dispersed via the digital light processing technology (Texas Instruments Inc., Dallas, TX, USA) which utilizes a digital micromirror device to project light within selected spectral bands onto a single element InGaAs detector.

#### 3.2.2. Data Acquisition

Tablet samples, before the HPLC analysis, were directly scanned through their blister. Spectra of ten tablets per batch were recorded for each formulation. Each spectrum was an average of six scans. 

#### 3.2.3. Chemometric Tools

##### Preprocessing

Spectral data were preprocessed using a Savitzky-Golay smoothing and differentiation filter (second-degree polynomial and second derivative) followed by an MSC. Chemometric analysis was performed on the spectral range between 1085 nm and 1601 nm.

##### Principal Component Analysis 

A PCA was performed on the collected AL spectra to have an overview of the distribution of the sample set.

##### DD-SIMCA

Class modeling methods such as SIMCA was used to evaluate the ability of the low-cost device to identify specifically the AL brand name. In this study, a data driven SIMCA (DD-SIMCA) has been employed. Similar to any SIMCA model, DD-SIMCA starts with the decomposition of the calibration spectra of the target class by PCA [10,35]. Then, the results of PCA decomposition is used to calculate a score distance (h_i_) and an orthogonal distance (v_i_) for each training sample. These distances are used to define the acceptance area or thresholds for the target class at a given significance level α. The DD-SIMCA results are most often shown using a two-dimensional plot through the coordinates ln (1 + h_i_/h_0_) vs. ln (1 + v_i_/v_0_), together with the limit/threshold curve which allows determining whether or not the samples belong to the target class [36]. The number of PCs mainly influences the quality of the classification and determines the complexity of the model. The model parameters (number of PCs and α) were optimized in a sequential way. Similar to any classification model, the performance of the DD-SIMCA model was assessed based on sensitivity and specificity. Sensitivity is related to the percentage of samples from the target class that are properly attributed as a member of the target class while specificity is the percentage of samples from non-members of the target class, which are properly attributed as non-members of the target class [36].

Three DD-SMICA models were built for the formulations for which at least five batches have been collected: Licit channel Combiart ^®^, Komefan^®^, and AL Macleods. Spectra of three batches were used as a training set to build the models and the two remaining batches were used as a test set to evaluate the model sensitivity. The other AL formulations were employed to mimic high quality fake drugs and test model specificity. All DD-SIMCA models were auto scaled.

#### 3.2.4. Software

Spectral preprocessing and PCA were carried out using the PLS_Toolbox version 8.2.1. while the DD-SIMCA analysis was done using DDSGUI, a graphical user interface freely available online [37]. All chemometric procedures were performed in a MATLAB environment (R2015a).

### 3.3. Procedure of HPLC-UV

#### 3.3.1. Instrumentation and Analytical Conditions

The HPLC analyses were carried out on the Dionex U3000 HPLC system (Thermo Scientific, Waltham, MA, USA) equipped with a pumping device, an autosampler, a column oven, and a diode array detector. The quaternary solvent delivery pump was able to work up to a pressure of 600 bars. The columns evaluated were J’sphere ODS-H80 (150 × 4.6 mm, 5 µm) from YMC CO., LTD (Kyoto, Japan), Symmetry C18 (150 × 3.0 mm, 5 µm), and XTerra RP18 (50 × 3.0 mm, 3.5 µm) both from Waters (Milford, MA, USA). The column temperature was maintained at 30 °C and UV detection performed at 210 nm. UV spectra from 200 nm to 400 nm were recorded for peak identification. The injection volume was 10 µL. An isocratic mobile phase containing EtOH 96% and 10 mM of acetic acid aqueous solution, pH 3.35 (60:40, *v*/*v*) was used at a flow rate of 0.5 mL/min. The quality of separation between artemether and lumefantrine was evaluated in different proportions of solvents and for each condition, retention factors (k), resolutions (Rs), and symmetry factors were calculated. The best conditions were achieved using the Symmetry C18 column and a mobile phase composed of EtOH 96% and 10 mM of acetic acid aqueous solution, pH 3.35 (63:37, *v*/*v*).

#### 3.3.2. Standard Sample Preparations

Stock solutions of artemether and lumefantrine were prepared at 1.0 and 1.2 mg/mL, respectively in EtOH 96% containing 0.5% of acetic acid which was added to improve the lumefantrine solubility. A mixture of artemether and lumefantrine at 0.1 and 0.6 mg/mL, respectively was prepared by diluting the appropriate volumes of stock solutions with the mobile phase. A solution of lumefantrine at 0.012 mg/mL was prepared by diluting the lumefantrine stock solution with the mobile phase.

#### 3.3.3. Validation

##### Specificity Study

Considering the excipients present in AL tablets, each excipient was prepared individually and in a mixture using EtOH 96% containing 0.5% of acetic acid, centrifuged, and the supernatant was injected to evaluate possible interfering peaks.

##### Linearity, Precision, and Accuracy

Validation was performed according to the total error approach based on the accuracy profile. Each day, stock solutions of lumefantrine and artemether were prepared at 1.2 and 1.0 mg/mL in 96% ethanol acidified with 0.5% of acetic acid. 

For the calibration set, one concentration level (100%) was used and consisted of 0.1 mg/mL for artemether and 0.012 mg/mL for lumefantrine. Calibration standards were prepared in duplicate by diluting appropriate volumes of stock solutions of each compound with the mobile phase.

For the validation set, three concentration levels (80–120%) were used for each compound: 0.08, 0.10, and 0.12 mg/mL for artemether, 0.0096, 0.0120, and 0.0144 mg/mL for lumefantrine. Based on excipients present in AL tablets and the amount commonly used, an excipient mixture composed of microcrystalline cellulose (36%), lactose (24%), maize starch (12%), hypromellose (4%), croscarmellose sodium (6%), crospovidone (6%), povidone (4%), talc (1%), magnesium stearate (2%), colloidal silica (1%), polysorbate 80 (2%), and sodium lauryl sulfate (2%) was prepared at a concentration of 5 mg/mL in ethanol acidified with 0.5% of acetic acid and was used as the matrix. Artemether validation sets were prepared by mixing appropriate volumes of artemether, lumefantrine stock solutions, and the excipient mixture and diluting with the mobile phase to reach the desired concentration of artemether, a concentration of 0.6 mg/mL for lumefantrine and a concentration of 0.5 mg/mL for the excipient mixture (excipients represent about 42% of the reconstituted form). Samples were centrifuged and the supernatant was injected. Validation standards were prepared in triplicate by repeating the dilution step. Lumefantrine validation sets were prepared by mixing appropriate volumes of lumefantrine, artemether stock solutions, and the excipient mixture and diluting with the mobile phase to reach the desired concentration of lumefantrine, a concentration of 0.002 mg/mL for artemether and a concentration of 0.01 the excipient mixture. Samples were centrifuged and the supernatant was injected. Validation standards were prepared in triplicate by repeating the dilution step.

All of this validation procedure was repeated at three different days.

#### 3.3.4. Tablet Sample Preparation

For each batch, five tablets were weighed and powdered. An appropriate amount of the powder was used to obtain a preparation containing lumefantrine at a concentration of about 1.2 mg (artemether at a concentration of about 0.2 mg/mL). Ethanol acidified with acetic acid (0.5%) was used as a dilution solvent. The obtained preparation was then centrifugated and the supernatant was used to perform two dilutions. The first dilution (1:2) with the mobile phase allowed reaching a concentration of 0.1 mg of artemether (0.6 mg of lumefantrine) and was used to determine the content of artemether in the tablets. The second dilution (1:100) allowed reaching a concentration of 0.012 mg/mL of lumefantrine and was used to determine the content of lumefantrine in the tablets.

## 4. Conclusions

The available results allow affirming that DD-SIMCA with a portable NIR spectrometer can be used as an analytical method for routine testing against pharmaceutical falsification of antimalarial artemether-lumefantrine drugs in their intact form and assisted with the quantitative analysis by the green RP-HPLC technique. These two analytical approaches are green and suitable for quality control of antimalarial artemether-lumefantrine tablets. They are complementary and correspond to a global analytical strategy which contributes to the fight against falsified medicines. The first approach consisted of evaluating the qualitative performance of a low-cost handheld NIR spectrophotometer associated to chemometric models in the detection of falsified drugs. Despite its limited spectral range and low resolution, the device allowed detecting falsified drugs with no active pharmaceutical ingredient and identifying specifically a brand name. This innovated handheld NIR spectrophotometer offers a promising performance and could be used as a first line screening tool in the detection and fight against falsified drugs particularly in developing countries. The second approach was quantitative and consisted of developing a green and fast RP-HPLC technique using ethanol as a green organic solvent and acetic acid as a green pH modifier coupled to the classical univariate calibration. The developed method was validated and applied to the analysis of AL tablets. This method allowed confirming that falsified drugs detected with the NIR instrument, did not contain an active pharmaceutical ingredient.

## Figures and Tables

**Table 1:** Artemether-lumefantrine tablets.

Brand Name	AL Dosage (mg)	Sales Channel	Test Batches
AL Ipca	20–120	Pharmacy	2
AL Macleods	20–120	Pharmacy	5
Artefan^®^	20–120	Pharmacy	2
Artefan^®^ Dispersible	20–120	Pharmacy	4
Combiart^®^	20–120	Pharmacy	6
Combiart^®^	20–120	Street vendors	3
Komefan^®^	20–120	Pharmacy	5

**Table 2:** Chromatographic parameters of artemether and lumefantrine according to the columns.

Columns	Artemether		Lumefantrin	
	Retention Factor (k)	Symmetry Factor (As)	Retention Factor (k)	Symmetry Factor (As)
Symmetry C18	4.60	1.10	2.78	1.22
J’sphere ODS-H80	6.47	1.16	3.40	1.75
Xterra RP18	2.50	1.31	4.48	1.28

**Table 3:** Eco-scale evaluation of the developed method.

Chemical Compound Score			Sub-Total PP	Total PP
Ethanol	Amount	<10–100 mL	2	2
	Hazard	Less severe hazard	1	
Water	Amount	<10–100 mL	2	0
	Hazard	none	0	
Acetic acid	Amount	<10 mL	1	1
	Hazard	1 pictogram-warming	1	
				∑ = 3
Instrument score				
				Total PP
Energy		≤0.1 kWh per sample		0
Occupational hazard		Analytical process hermetization		0
Waste	1–10 mL			3
				∑ = 3
Total penalty points (PP): 6				
Analytical Eco-scale score: 100 − 6 = 94				

**Table 4:** Validation results of artemether and lumefantrine.

Validation Criteria		Artemether		Lumefantrine	
Linearity	Slope	0.9986		1.0342	
	Intercept	0.0028		0.0001	
	R^2^	0.992		0.993	
Trueness	Level	Concentration (mg/mL)	Relative Bias (%)	Concentration (mg/mL)	Relative Bias (%)
	80%	0.08	5.03	0.0096	5.22
	100%	0.10	−0.08	0.0120	1.72
	120%	0.12	0.77	0.0144	0.05
Precision	Level	Repeatability (RSD%)/Intermediate Precision (RSD%)		Repeatability (RSD%)/Intermediate Precision (RSD%)	
	80%	2.30/2.40		1.75/1.87	
	100%	1.33/2.86		2.36/3.36	
	120%	1.18/3.10		0.99/0.99	
Accuracy	Level	β-Expectation Tolerance Limits (%)		β-Expectation Tolerance Limits (%)	
	80%	[0.62, 9.43]		[1.75, 8.69]	
	100%	[−6.30, 6.15]		[−4.92, 8.37]	
	120%	[−6.40, 7.93]		[−1.77, 1.87]	

**Table 5:** Results of the HPLC analysis of AL tablets.

Brand Name	Tested Batches	Artemether Content (%) Range	Lumefantrine Content (%) Range
AL Ipca	2	94.5–99.5	96.7–98.7
AL Macleods	5	94.6–104.4	97.5–106.0
Artefan^®^	2	98.9–99.6	99.6–101.4
Artefan^®^ Dispersible	4	97.0–101.2	98.4–101.6
Komefan^®^	5	95.6–98.8	97.8–100.9
Licit channel Combiart^®^	6	95.8–106.0	98.4–102.3
Illicit channel Combiart^®^	3	Not detected	Not detected
